# Effects of Angiopoietin-Like 3 on Triglyceride Regulation, Glucose Homeostasis, and Diabetes

**DOI:** 10.1155/2019/6578327

**Published:** 2019-03-03

**Authors:** Eliza Christopoulou, Moses Elisaf, Theodosios Filippatos

**Affiliations:** ^1^Department of Internal Medicine, School of Medicine, University of Ioannina, Ioannina, Greece; ^2^Department of Internal Medicine, School of Medicine, University of Crete, University Hospital of Heraklion, Heraklion, Crete, Greece

## Abstract

Angiopoietin-like 3 (ANGPTL3) is a regulator of plasma triglyceride (TRG) levels due to its inhibitory action on the activity of lipoprotein lipase (LPL). ANGPTL3 is proteolytically cleaved by proprotein convertases to generate an active N-terminal domain, which forms a complex with ANGPTL8 orchestrating LPL inhibition. ANGPTL3-4-8 mouse model studies indicate that these three ANGPTL family members play a significant role in partitioning the circulating TRG to specific tissues according to nutritional states. Recent data indicate a positive correlation of ANGPTL3 with plasma glucose, insulin, and homeostatic model assessment of insulin resistance (HOMA-IR) in insulin-resistant states. The aim of this review is to critically present the metabolic effects of ANGPTL3, focusing on the possible mechanisms involved in the dysregulation of carbohydrate homeostasis by this protein. Heterozygous and homozygous carriers of ANGPTL3 loss-of-function mutations have reduced risk for type 2 diabetes mellitus. Suggested mechanisms for the implication of ANGPTL3 in carbohydrate metabolism include the (i) increment of free fatty acids (FFAs) owing to the enhancement of lipolysis in adipose tissue, which can induce peripheral as well as hepatic insulin resistance; (ii) promotion of FFA flux to white adipose tissue during feeding, leading to the attenuation of de novo lipogenesis and decreased glucose uptake and insulin sensitivity; (iii) induction of hypothalamic LPL activity in mice, which is highly expressed throughout the brain and is associated with enhanced brain lipid sensing, reduction of food intake, and inhibition of glucose production (however, the effects of ANGPTL3 on hypothalamic LPL in humans need more clarification); and (iv) upregulation of ANGPTL4 expression (owing to the plasma FFA increase), which possibly enhances insulin resistance due to the selective inhibition of LPL in white adipose tissue leading to ectopic lipid accumulation and insulin resistance. Future trials will reveal if ANGPTL3 inhibition could be considered an alternative therapeutic target for dyslipidemia and dysglycemia.

## 1. Introduction

Recently, a specific family of secretory proteins has been named “angiopoietin-like proteins” (ANGPTLs) due to their structural similarity to angiopoietins, the key factors that regulate angiogenesis [1]. Angiopoietin-like 3 (ANGPTL3) is a 70 kDa protein, and although it does not bind to the receptor tyrosine kinase Tie2 like the angiopoietin family proteins, it induces angiogenesis by binding to integrin *α*_v_*β*_3_ [2]. However, ANGPTL3 has mainly emerged as an important regulator of plasma triglyceride (TRG) levels due to its inhibitory action on the activity of lipoprotein lipase (LPL), the enzyme which is attached to the capillary endothelium and catalyses the hydrolytic cleavage of TRG into fatty acids [3]. Interestingly, recent data show a positive correlation of ANGPTL3 with plasma glucose, insulin, and homeostatic model assessment of insulin resistance (HOMA-IR) in insulin-resistant states, implying that ANGPTL3 may also participate in glucose homeostasis. The aim of this review is to critically present the metabolic effects of ANGPTL3, focusing on the possible mechanisms involved in the dysregulation of carbohydrate homeostasis by this protein.

## 2. ANGPTL3 Regulation

ANGPTL3 is exclusively produced in the liver and can therefore be classified as a true hepatokine [4, 5]. It is released into the circulation where it undergoes cleavage by hepatic proprotein convertases. This cleavage has been shown to be essential for the activation of ANGPTL3 [6]. The main factors that have been reported to downregulate ANGPTL3 mRNA expression *in vivo* and/or *in vitro* include insulin [4, 7, 8], leptin [7], peroxisome proliferator-activated receptor- (PPAR-) *β* [9], statins [10], and thyroid hormone [11, 12]; in contrast, the liver X receptor (LXR) upregulates ANGPTL3 mRNA expression [13].

## 3. ANGPTL3 and Lipid Metabolism (Figure 1)

ANGPTL3 is an important regulator of LPL, which is a key enzyme in the lipolysis of TRG of very low-density lipoproteins (VLDL) and chylomicrons [3, 14, 15]. After hydrolysis of TRG by LPL, the remnants of chylomicrons [16] and VLDL [17] are cleared via specific hepatic receptors, while the remaining free fatty acids (FFAs) are taken up by peripheral tissues as sources of energy [18]. This process plays an important role in lipid metabolism. ANGPTL3 decreases LPL activity [19–22]; thus, animals overexpressing ANGPTL3 manifest hypertriglyceridemia [23]. Accordingly, mice lacking ANGPTL3 have increased LPL activity and reduced levels of TRG and FFA [19, 22, 24]. Loss-of-function (LOF) mutations in the ANGPTL3 gene have been also linked to a rare recessive disorder termed familial combined hypobetalipoproteinemia (FHBL2), which is characterized by decreased serum levels of TRG, high-density lipoprotein (HDL) cholesterol, and low-density lipoprotein (LDL) cholesterol [24, 25].

ANGPTL3 is proteolytically cleaved by proprotein convertases to generate an active N-terminal domain that seems to inhibit LPL [6]. It has been shown that angiopoietin-like 8 (ANGPTL8) interacts with ANGPTL3 and enhances ANGPTL3 cleavage, releasing the N-terminal domain [26]; then, ANGPTL8 and the N-terminal of ANGPTL3 form a complex that orchestrates LPL inhibition [26–28]. Recently, an ANGPTL3-4-8 model suggested that these three ANGPTL family members play a significant role in partitioning the circulating TRG to specific tissues according to nutritional states [18, 29]. Particularly, during feeding, ANGPTL8 levels are increased and activate the ANGPTL8-ANGPTL3 pathway, which inhibits LPL in cardiac and skeletal muscles [30], thereby making circulating TRG available for uptake by white adipose tissue (WAT), in which LPL activity is elevated owing to diminished ANGPTL4. The reverse is present during fasting, which suppresses ANGPTL8 but induces ANGPTL4, directing TRG to muscles [18, 29].

Apart from its role as an LPL inhibitor, ANGPTL3 affects lipid metabolism by additional mechanisms. Firstly, it interferes with endothelial lipase (EL) activity *in vitro* [31]; accordingly, LOF mutations of ANGPTL3 are associated with low levels of HDL cholesterol [24]. Moreover, ANGPTL3 induces lipolysis in adipose tissue, leading to the release of FFA and glycerol from adipocytes [32]. The supply of FFA from adipocytes to the liver results in an increase in hepatic VLDL synthesis [33]. Supporting this hypothesis, *in vivo* kinetic studies have shown that FHBL2 individuals have decreased production rates of VLDL by the liver [24]. Additionally, the low LDL cholesterol levels observed in families with FHBL2 [24] could be partially explained from the decreased synthesis and secretion of VLDL from the liver since, following lipolysis by LPL, VLDL and its metabolic intermediate, IDL, have two possible fates: clearance by the liver or further metabolism to LDL [34]. Also, in a recent study, it was shown that ANGPTL3 silencing results in the enhancement of VLDL/LDL uptake [35]. This could be explained via the higher lipolytic activity of LPL in the context of ANGPTL3 silencing, leading to a change in the content of TRG in the LDL/VLDL particles, which may be cleared more rapidly [35]. Taken together, enhanced ANGPTL3 activity correlates positively with increased serum TRG, LDL cholesterol, and HDL cholesterol levels.

## 4. ANGPTL3 and Glucose Homeostasis (Figure 2)

Increased levels of ANGPTL3 are observed in subjects with type 2 diabetes mellitus (T2DM) in comparison with nondiabetic subjects [8, 36]. Additionally, obese nondiabetic subjects exhibited significantly increased plasma ANGPTL3 levels compared with nonobese nondiabetic subjects [36]. Moreover, plasma insulin, glucose, and HOMA-IR were significantly lower in homozygous subjects with a LOF ANGPTL3 mutation compared with heterozygotes and noncarriers [37]. Accordingly, in a cross-sectional study, ANGPTL3 concentration was independently associated with insulin resistance (assessed by the HOMA-IR) [38]. All these data support the notion that ANGPTL3 is elevated in insulin-resistant states and may interfere with carbohydrate metabolism.

Suggested mechanisms for the ANGPTL3-induced deterioration of carbohydrate metabolism include the following.

### 4.1. ANGPTL3 Induces Insulin Resistance through Enhancement of Lipolysis

A possible mechanism of ANGPTL3-induced deterioration of glucose metabolism is the increment of FFA owing to the enhancement of lipolysis in adipose tissue [32], which can induce peripheral as well as hepatic insulin resistance [39, 40]. Notably, fatty acid metabolites in skeletal muscles activate a serine/threonine kinase cascade by protein kinase C*θ* (PKC*θ*), which results in a lessened phosphorylation of insulin receptor substrate-I (IRS-I) and in turn in reduced phosphatidylinositol-3-OH (PI-3) kinase activation [39, 41]. Consequently, glucose transport activity in skeletal muscles is decreased. Accordingly, in the liver, the elevated FFA levels attenuate the phosphorylation of IRS-2 and diminish the hepatic insulin signaling [42]. This leads to a lessened suppression of glycogenolysis by insulin [40] and enhancement of gluconeogenesis [43], thereby inducing insulin resistance.

It has been shown that serum levels of ANGPTL3 are significantly increased in the setting of the more advanced forms of nonalcoholic fatty liver disease (NAFLD) (definite and borderline nonalcoholic steatohepatitis (NASH)), but not in patients with fatty liver [38]. Of note, ANGPTL3 levels above the cutoff value of 400.5 ng/ml were significantly and independently associated with the presence of definite NASH after adjusting for potential confounders, including age, sex, body mass index (BMI), HOMA-IR, aspartate transaminase (AST), and alanine transaminase (ALT) [38]. Additionally, a strong relationship was found between ANGPTL3 and cytokeratin 18 (CK-18), a protein associated with apoptotic cell death of hepatocytes [44]. Several studies have demonstrated the elevation of CK-18 in the context of NASH and hepatic inflammation [45]. Since hepatic insulin resistance and T2DM have been considered sequelae of NAFLD [42], ANGPTL3 could possibly affect glucose levels by inducing NAFLD through lipolysis and increased FFA flux to the liver [42].

### 4.2. ANGPTL3 Attenuates De Novo Lipogenesis Leading to Diminished Insulin Sensitivity

During feeding, ANGPTL3 promotes the flux of FFA to white adipose tissue to replenish TRG stores that are depleted during fasting [29]. In ANGPTL3-knockout mice, the uptake of FFA to white adipose tissue was abolished and led to an increment of glucose uptake [29]. In turn, glucose uptake induces and activates the nuclear transcription factor carbohydrate response element-binding protein (ChREBP), which enhances lipogenesis [29, 46]. Increased de novo lipogenesis in white adipose tissue has been correlated with insulin sensitivity [47]. Hence, it is likely that the increased uptake of glucose into white adipose tissue and the subsequent increment of de novo lipogenesis explain the increased insulin sensitivity associated with the inactivation of ANGPTL3. ChREBP enhances also the synthesis of the recently discovered branched fatty acid esters of hydroxyl fatty acids (FAHFAs), a family of a novel class of lipids [47]. FAHFAs enhance the insulin-mediated glucose uptake in adipocytes and augment glucose-stimulated glucagon-like protein (GLP)-1 secretion from enteroendocrine cells and insulin secretion by pancreatic beta cells [47, 48].

### 4.3. ANGPTL3 Induces Hypothalamic LPL Activity in Mice

The inhibition of hypothalamic LPL could be considered another plausible mechanism of ANGPTL3-induced deterioration of carbohydrate homeostasis. LPL is highly expressed throughout the brain, including the hypothalamus and hippocampus [49]. Increased LPL activity is associated with promotion of lipid uptake by hypothalamic neurons, leading to enhanced brain lipid sensing [50, 51]. In particular, the accumulation of fatty acid-derived long-chain fatty acids (LCFAs) results in a reduction of food intake and inhibition of glucose production [51]. ANGPTL3 is also highly expressed in the neurons of the mediobasal hypothalamus, a brain area critical for the control of energy balance [52]. However, intracerebroventricular (ICV) administration of ANGPTL3 significantly stimulated hypothalamic LPL activity in mice [52], whereas the inhibition of hypothalamic ANGPTL3 suppressed hypothalamic LPL activity [52]. These findings are opposite to the inhibitory effects of ANGPTL3 on peripheral LPL. Intriguingly, ICV injection of ANGPTL3 in mice suppressed fasting-induced feeding in a dose-dependent manner and inhibited weight gain [52]. However, more evidence is needed to establish the impact of ANGPTL3 on hypothalamic LPL activity in humans.

### 4.4. ANGPTL3 Upregulates ANGPTL4 Expression through the Elevation of Plasma FFAs

As mentioned above, ANGPTL3 increases plasma FFA levels due to enhancement of lipolysis. In turn, FFAs upregulate the expression of ANGPTL4 through the activation of PPAR-*α* in the liver [53], PPAR-*γ* in the white adipose tissue [53, 54], and PPAR-*δ* in the myocytes [55–57] and macrophages [58]. Published data do not present a uniform picture on the influence of ANGPTL4 on glucose metabolism and insulin sensitivity [52]. In particular, ANGPTL4 correlates positively with glucose levels [59, 60], and it was higher among individuals with T2DM [36, 61], as well as in obese nondiabetic humans compared with nonobese nondiabetic subjects [36]. Accordingly, LOF alleles of ANGPTL4 were associated with a lower risk of T2DM [62]. However, in a study of Xu et al., ANGPTL4 levels were lower in T2DM patients compared with nondiabetic subjects, and overexpression of ANGPTL4 in mice decreased blood glucose levels and improved glucose tolerance, possibly via reduced hepatic glucose production [63]. Indeed, infection of primary hepatocytes with ANGPTL4 adenovirus significantly decreased secretion of glucose into the medium, suggesting that ANGPTL4 may lower plasma glucose by decreasing hepatic glucose output [64]. At the same time, ANGPTL4 overexpression was associated with a decrease in insulin-mediated glucose disposal, suggesting peripheral insulin resistance [64]. In accordance with the above, in clamp studies, whole-body transgenic ANGPTL4 overexpression led to impaired glucose utilisation and insulin resistance in the periphery and higher insulin-mediated suppression of glucose production in the liver [64].

The enhancement of insulin resistance in the context of elevated ANGPTL4 levels could be explained by the selective inhibition of LPL in white adipose tissue, which leads to an ectopic lipid accumulation and insulin resistance [65]. However, the rerouting of TRG into the heart and skeletal muscle might constitute a positive mechanism as it increases energy influx into oxidative tissues [65].

It is of great interest that circulating ANGPTL4 in humans is affected by obesity status and altered glucose tolerance, as well as by long-term body weight changes [60, 65]. In a recent cross-sectional study, plasma ANGPTL4 levels were increased in obese patients compared to lean individuals, and this elevation was more pronounced in obese subjects with altered glucose tolerance [60]. Also, ANGPTL4 was positively correlated with obesity-associated characteristics such as BMI, waist circumference, and fat mass, as well as with altered glucose tolerance-associated hallmarks (glycated haemoglobin (HbA1c), HOMA-IR, and fasting TRG) [60, 65]. Notably, plasma ANGPTL4 levels significantly increased after weight gain and decreased after weight loss [60]. In addition, an increase in plasma ANGPTL4 throughout pregnancy was positively associated with gestational weight gain and could be used as an early marker of increased susceptibility to excess gestational weight gain [66].

A recent study of Janssen et al. showed that ANGPTL4 uncouples visceral obesity from glucose intolerance partly via the gut microbiota [67]. Particularly, ANGPTL4 loss, although it leads to an increment of visceral adipose tissue mass, also raises plasma insulin levels and reduces glucose intolerance via a mechanism that is at least partly dependent on the gut microbiota [67]. Accordingly, suppression of the gut bacteria using antibiotics abolished the increased glucose tolerance in the ANGPTL4-deficient mice [67]. Of note, the improved glucose tolerance in ANGPTL4-deficient mice was accompanied by elevated insulin levels but not increased insulin sensitivity, suggesting that the lower plasma glucose levels are caused by increased insulin secretion [67]. In accordance with the above observations, transfer of faecal microbiota from lean donors to recipients with the metabolic syndrome increased insulin sensitivity [68], but more research is needed for the delineation of the involved mechanisms.

In addition, recent studies showed that hypothalamic ANGPTL4 is engaged in the regulation of feeding behaviour and body weight. Specifically, ICV administration of ANGPTL4 in mice suppresses food intake and decreases body weight [69]. Accordingly, in ANGPTL4-deficient mice, food intake following a fast was significantly greater and was normalized by centrally administered ANGPTL4 [69].

## 5. Discussion

Glucose intolerance and hypertriglyceridemia are common aspects of metabolic syndrome and are considered as risk factors for the development of T2DM and atherosclerotic cardiovascular disease [70, 71]. Heterozygous and homozygous carriers of ANGPTL3 LOF mutations have reduced risk for T2DM and coronary heart disease [72, 73]. Moreover, in healthy human subjects, ANGPTL3 levels showed a positive association with carotid artery intima-media thickness and femoral artery intima-media thickness independent of age, sex, smoking, BMI, systolic blood pressure, insulin resistance index, TRG, glucose, LDL cholesterol, and HDL cholesterol levels [74].

In aggregate, these data indicate that ANGPTL3 inhibition could be considered an alternative therapeutic target for dyslipidemia, dysglycemia, and possible reduction of atherosclerotic lesion size. Pharmacological inhibition of ANGPTL3 was assessed in preclinical and phase I clinical trials. Notably, dyslipidemic mice treated with evinacumab, a human monoclonal antibody inhibitor of ANGPTL3, exhibited, on top of TRG, LDL cholesterol and HDL cholesterol levels reduction, a significant decrease in the atherosclerotic lesion area, similar to that reported in the same mouse model treated with atorvastatin [73]. Moreover, mice treated with antisense oligonucleotides (ASOs) targeting ANGPTL3 messenger RNA (mRNA) manifested dose-dependent reductions in LDL cholesterol, TRG, atherosclerosis progression, and liver TRG content and an increase in insulin sensitivity [75]. The phase I trials on healthy volunteers strengthened these results, with a significant reduction of TRG, LDL cholesterol, and HDL cholesterol levels [73, 75].

## 6. Conclusions

Plasma ANGPTL3 levels are positively correlated with TRG levels but are also increased in insulin-resistant states indicating a possible implication of ANGPTL3 in carbohydrate metabolism. This could be partially explained by the elevation of FFAs and the subsequent hepatic and peripheral insulin resistance due to ANGPTL3-induced lipolysis. Moreover, the enhanced flux of FFAs to white adipose tissue during feeding in the context of elevated ANGPTL3 levels is associated with diminished de novo lipogenesis and a reduction in insulin sensitivity. Also, the increment of FFA levels results in an increment of ANGPTL4 expression, which also seems to be implicated in glucose metabolism. The effects of ANGPTL3 on hypothalamic LPL activity need more clarification since it may play an important role on food intake and glucose production. The role of ANGPTL3 on carbohydrate metabolism may be important; however, it has not yet been well confirmed by experimental studies, and it is based mainly on correlations between levels of ANGPTL3 and insulin sensitivity/resistance or weight loss/gain. The conduct of more studies is needed in order to determine this connection and to assess the possibility that the pharmacological inhibition of ANGPTL3 may become a future treatment option for the improvement of dysglycemia.

## Figures and Tables

**Figure 1:**
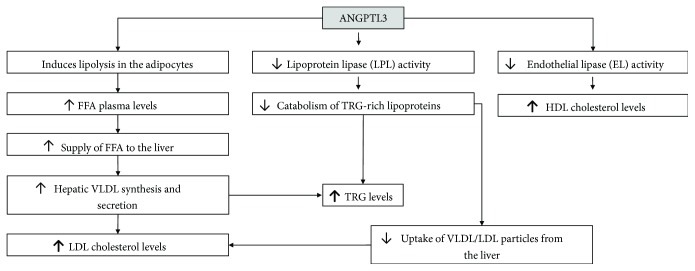
Effects of angiopoietin-like 3 (ANGPTL3) on lipoprotein metabolism. FFA: free fatty acid; TRG: triglycerides; HDL: high-density lipoprotein; LDL: low-density lipoprotein; VLDL: very low-density lipoprotein.

**Figure 2:**
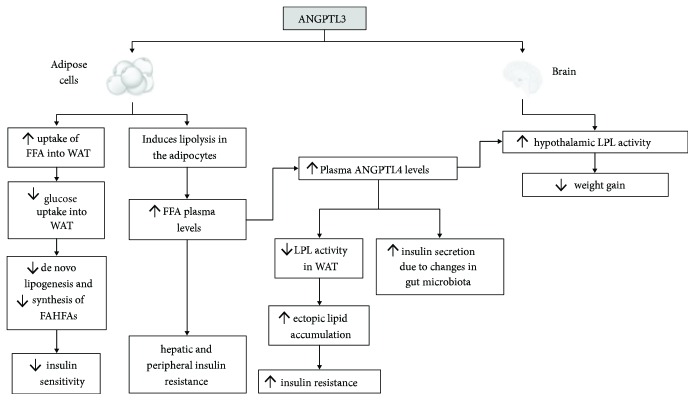
Effects of angiopoietin-like 3 (ANGPTL3) on carbohydrate metabolism. FFA: free fatty acid; WAT: white adipose tissue; FAHFAs: fatty acid esters of hydroxyl fatty acids; ANGPTL4: angiopoietin-like 4; LPL: lipoprotein lipase.
